# Coral-Associated Bacterial Diversity Is Conserved across Two Deep-Sea *Anthothela* Species

**DOI:** 10.3389/fmicb.2016.00458

**Published:** 2016-04-05

**Authors:** Stephanie N. Lawler, Christina A. Kellogg, Scott C. France, Rachel W. Clostio, Sandra D. Brooke, Steve W. Ross

**Affiliations:** ^1^College of Marine Science, University of South Florida, St. PetersburgFL, USA; ^2^U.S. Geological Survey, St. Petersburg Coastal and Marine Science Center, St. PetersburgFL, USA; ^3^Department of Biology, University of Louisiana at LafayetteLafayette, LA, USA; ^4^Coastal and Marine Laboratory, Florida State University, St. TeresaFL, USA; ^5^Center for Marine Science, University of North Carolina WilmingtonWilmington, NC, USA

**Keywords:** cold-water corals, deep sea, bacteria, octocoral, gorgonian, submarine canyons, microbiome

## Abstract

Cold-water corals, similar to tropical corals, contain diverse and complex microbial assemblages. These bacteria provide essential biological functions within coral holobionts, facilitating increased nutrient utilization and production of antimicrobial compounds. To date, few cold-water octocoral species have been analyzed to explore the diversity and abundance of their microbial associates. For this study, 23 samples of the family Anthothelidae were collected from Norfolk (*n* = 12) and Baltimore Canyons (*n* = 11) from the western Atlantic in August 2012 and May 2013. Genetic testing found that these samples comprised two *Anthothela* species (*Anthothela grandiflora* and *Anthothela* sp.) and *Alcyonium grandiflorum*. DNA was extracted and sequenced with primers targeting the V4–V5 variable region of the 16S rRNA gene using 454 pyrosequencing with GS FLX Titanium chemistry. Results demonstrated that the coral host was the primary driver of bacterial community composition. *Al. grandiflorum*, dominated by Alteromonadales and Pirellulales had much higher species richness, and a distinct bacterial community compared to *Anthothela* samples. *Anthothela* species (*A. grandiflora* and *Anthothela* sp.) had very similar bacterial communities, dominated by Oceanospirillales and Spirochaetes. Additional analysis of core-conserved bacteria at 90% sample coverage revealed genus level conservation across *Anthothela* samples. This core included unclassified Oceanospirillales, Kiloniellales, Campylobacterales, and genus *Spirochaeta*. Members of this core were previously recognized for their functional capabilities in nitrogen cycling and suggest the possibility of a nearly complete nitrogen cycle within *Anthothela* species. Overall, many of the bacterial associates identified in this study have the potential to contribute to the acquisition and cycling of nutrients within the coral holobiont.

## Introduction

Cold-water coral ecosystems contribute to vital biodiversity hotspots within the deep sea. Thriving in temperatures that range from 4 to 12°C, these corals are globally distributed, occurring at depths between 50 and 4,000 m and inhabiting locations with strong currents and elevated topography (e.g., continental slopes and seamounts; Roberts and Hirshfield, 2004; White et al., 2005; Roberts et al., 2006). Similar to tropical reefs (Rohwer et al., 2002; Bourne and Munn, 2005), cold-water ecosystems provide a critical habitat for many organisms (Roberts et al., 2006) and are home to diverse and complex microbial assemblages (Penn et al., 2006; Neulinger et al., 2008, 2009; Hansson et al., 2009; Kellogg et al., 2009; Galkiewicz et al., 2011; Gray et al., 2011; Bourne and Webster, 2013). Research addressing the microbial communities associated with cold-water corals has been limited due to the expense of sampling, which can be directly linked to the difficulty of sample retrieval at depth. While many of these corals have been identified since the 1800s, the first microbial study of cold-water corals was not published until 2006. This study assessed microbiota associated with dead and living samples of the scleractinian coral *Lophelia pertusa* (Yakimov et al., 2006). That same year, Penn et al. (2006) evaluated bacterial communities associated with a black coral and several bamboo corals in the Gulf of Alaska. These two studies were the first to describe the microbial communities associated with stony and soft cold-water coral species as well as demonstrate differentiation between these deep-sea coral-associated communities and those of their surrounding environments (sediment and water column). Since then, studies have characterized the microbial diversity of additional cold-water corals: *L. pertusa* (Kellogg, 2008; Neulinger et al., 2008; Hansson et al., 2009; Kellogg et al., 2009; Schottner et al., 2009; Galkiewicz et al., 2011), *Madrepora oculata* (Hansson et al., 2009), and octocorals *Paragorgia arborea*, *Plumarella superba*, and *Cryogorgia koolsae* (Gray et al., 2011).

Previous studies have examined bacterial function within the coral host microbiomes. While some bacteria appear to play commensal or pathogenic roles (Nissimov et al., 2009; Shnit-Orland and Kushmaro, 2009; Bourne and Webster, 2013), many are not static in function, fluctuating with transitioning environmental conditions [e.g., increased microbial pathogenicity upon exposure to elevated thermal stressors (Bruno et al., 2007; Ben-Haim et al., 2003a)]. Because corals are dependent (in part) on their microbe–host interactions, examining the “core conserved” communities may reveal insights into the overall health of the coral host (Shade and Handelsman, 2012; Krediet et al., 2013). Many variables influence the bacterial presence within the coral holobiont including: specificity to host (genus; Littman et al., 2009, or species; Rohwer et al., 2002), association within a host niche (e.g., tissue vs. mucus; Bourne and Munn, 2005; Koren and Rosenberg, 2006; Sweet et al., 2010; Ainsworth et al., 2015), or fluctuations in the surrounding environment (Pantos et al., 2003; Reshef et al., 2006; Ainsworth and Hoegh-Guldberg, 2009). While bacterial communities may vary based on these parameters, conserved bacteria necessary for coral host health, defined as the “core” community, are consistently present. Because little is known about the bacterial functions within the cold-water coral holobiont, it is necessary to identify the core microbiota of each coral species (defined as those common in more than one sample; Shade and Handelsman, 2012).

Because cold-water corals are azooxanthellate (they do not acquire nutrients through photosynthesis), they obtain their nutrients from floating particulates and planktonic organisms and the microbial functional potential expressed within the coral holobiont (White et al., 2005; Zhang et al., 2015). Understanding the metabolic pathways and biochemical processing of nutrients such as nitrogen, carbon, and sulfur are essential in coral health and development (Zhang et al., 2015). Based on phylogenetic inference and functional gene data, nitrogen-cycling microbes appear to be common members of tropical stony coral microbiomes (Wegley et al., 2007; Olson et al., 2009; Kimes et al., 2010; Lema et al., 2012, 2014; Olson and Lesser, 2013; Pratte, 2013; Yang et al., 2013, 2015) and have been suggested to be species-specific (Wafar et al., 1990; Lema et al., 2012). The process of nitrogen fixation has been detected in a number of tropical stony corals (Williams et al., 1987; Shashar et al., 1994; Grover et al., 2014; Rädecker et al., 2014), as has nitrification (Wafar et al., 1990). Most nitrogen cycling studies have been conducted on stony corals, but there are a couple of recent studies that provide evidence of similar activities (nitrogen fixation, nitrite reduction, and ammonia oxidation) in soft corals (Yang et al., 2013; Bednarz et al., 2015). A recent study has proposed that nitrogen cycling might play a significant role in supplementing nutrition within the cold-water coral, *L. pertusa* (Middelburg et al., 2015). While it is evident that nitrogen availability is one of the driving factors in the proliferation and health of tropical coral hosts, little is known about its influence in the cold-water coral holobionts.

In an effort to further our understanding of cold-water octocorals and their microbial associates, this study evaluated three corals from the family Anthothelidae, initially targeting the species *Anthothela grandiflora*. Endemic to the Atlantic Ocean, *A. grandiflora* was first observed off the coast of Nova Scotia in the mid-1800s (Whiteaves, 1901), but to date no microbial analysis has been completed. For this study, samples from 23 individual colonies of gorgonian corals visually identified as *Anthothela* were collected from Baltimore and Norfolk canyons off the east coast of the United States in the Mid-Atlantic Bight. Overall, our objective was to provide the first characterization of the bacterial diversity associated with the cold-water octocoral genus *Anthothela*. Based on tropical coral research, we hypothesized that (1) bacterial communities would be conserved at the species level (Rohwer et al., 2002), (2) that different canyons might influence the bacterial community composition (Littman et al., 2009; Barott et al., 2011), and (3) that nitrogen-cycling microbes would be an important part of *Anthothela* spp. holobionts and constitute part of the core microbiome (Lema et al., 2012, 2014; Rädecker et al., 2015).

## Materials and Methods

### Sample Sites and Collections

**FIGURE 1:**
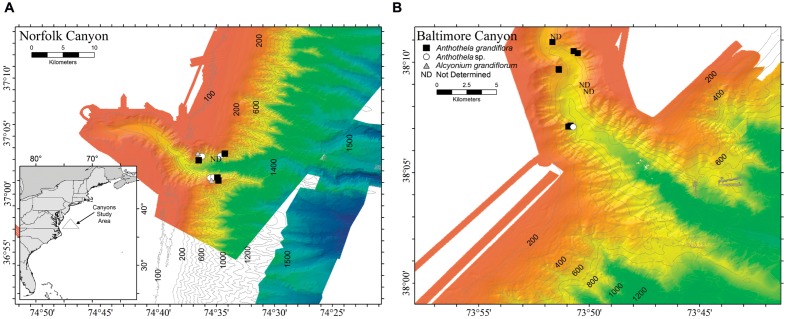
**Map of collection sites.** Samples were collected from two submarine canyons, **(A)** Norfolk and **(B)** Baltimore, located off the Mid-Atlantic coast of the United States. Symbol shapes defined in the legend distinguish the coral samples based on genetic identification (e.g., *Anthothela grandiflora*, *Anthothela* sp., and *Alcyonium grandiflorum).*

In total, 23 individual Anthothelidae colonies were sampled during two research cruises conducted in August 2012 and May 2013. Site locations in the Mid-Atlantic Bight included Baltimore Canyon, which was sampled using the *Kraken II* remotely-operated vehicle (ROV; University of Connecticut) in 2012 and Norfolk Canyon sampled using the *Jason II* ROV (Woods Hole Oceanographic Institution) in 2013 (**Figure 1**). Environmental parameters were recorded for each sample site including location (latitude and longitude), depth, temperature, and salinity (**Table 1**). Depths of sample collection ranged from 401 to 704 m. Bottom types varied from rocky seafloor to rock cliffs and ledges. Common benthic fauna present at sample sites associated with coral colonies included adult galatheid squat lobsters (*Eumunida picta*) and cutthroat eels (*Synaphobranchidae*). Several scleractinians and octocorals were present in the vicinity of Anthothelidae colonies, including *Desmophylum*, *Paragorgia*, and *Primnoa* species.

**Table 1:** Sample collection and corresponding environmental data.

Coral	Year	Canyon	ID #	Latitude	Longitude	Depth (m)	Temp (°C)	Salinity (psu)
*Anthothela grandiflora*	2012	Baltimore	NF.13Q6	38.161487	-73.856465	434	7.1	35.1
*Anthothela grandiflora*	2012	Baltimore	NF.13Q7	38.161070	-73.856163	432	7.5	35.1
*Anthothela grandiflora*	2012	Baltimore	NF.15Q6	38.173512	-73.841965	416	7.2	35.1
*Anthothela grandiflora*	2012	Baltimore	NF.15Q7	38.175122	-73.845125	457	6.8	35.1
*Anthothela grandiflora*	2012	Baltimore	NF.16Q7	38.181985	-73.861092	436	6.3	35.0
*Anthothela grandiflora*	2012	Baltimore	NF.17Q6	38.118832	-73.847417	575	5.7	35.0
*Anthothela grandiflora*	2012	Baltimore	NF.17Q7	38.118210	-73.847688	575	5.7	35.0
*Anthothela grandiflora*	2012	Baltimore	NF.18Q7	38.117922	-73.845465	679	5.1	35.0
*Anthothela grandiflora*	2013	Norfolk	RB.686Q4	37.054690	-74.603935	581	5.9	35.0
*Anthothela grandiflora*	2013	Norfolk	RB.687Q5	37.054808	-74.578777	606	5.7	35.0
*Anthothela grandiflora*	2013	Norfolk	RB.688Q1	37.024297	-74.588163	559	5.8	34.9
*Anthothela grandiflora*	2013	Norfolk	RB.688Q5	37.024247	-74.588199	560	5.8	34.9
*Anthothela* sp.	2012	Baltimore	NF.18Q6	38.1181583	-73.849030	524	5.5	35.0
*Anthothela* sp.	2013	Norfolk	RB.686Q5	37.054699	-74.603939	581	5.9	35.0
*Anthothela* sp.	2013	Norfolk	RB.687Q3	37.054881	-74.577786	594	5.6	35.0
*Anthothela* sp.	2013	Norfolk	RB.688Q2	37.023429	-74.592413	474	6.5	35.0
*Anthothela* sp.	2013	Norfolk	RB.688Q4	37.024201	-74.588153	559	5.8	34.9
*Alcyonium grandiflorum*	2013	Norfolk	RB.686Q2	37.058587	-74.605852	480	6.6	35.1
*Alcyonium grandiflorum*	2013	Norfolk	RB.688Q3	37.023538	-74.592445	474	6.4	35.0
ND*	2012	Baltimore	NF.01Q7	38.149448	-73.837895	451	6.4	35.1
ND*	2012	Baltimore	NF.02Q7	38.144950	-73.834483	401	6.8	35.1
ND*	2012	Baltimore	NF.16Q6	38.181962	-73.860835	435	5.7	35.0
ND*	2013	Norfolk	RB.687Q4	37.053907	-74.580567	704	5.3	35.0

Highlighted samples (*n* = 16) were used in final analysis. *Samples listed as ND did not have genetic analysis completed.

Because some species of Anthothelidae can grow over other organisms, including dead coral branches and sponges, great care was taken to select sections of polyps from the tips of branches rather than the main stalk (Lumsden et al., 2007). This technique was employed to avoid accidental contamination of the sampled coral microbiome with that of the supporting organism. Branches were removed using the ROV’s manipulator claw and each sample placed in an individual polyvinyl chloride (PVC) quiver. The quivers were cleaned before deployment using ethanol to remove any interior biofilms, filled with freshwater and sealed with a rubber stopper. This prevented contamination of the containers by the water column prior to sampling, during which the rubber stopper is removed and *in situ* seawater would replace the freshwater due to density differences. On the ship, coral samples were transferred from the ROV collection quivers to sterile 50 mL tubes containing the preservative RNAlater (Life Technologies, Grand Island, NY, USA). Samples were incubated overnight at 4°C to allow the preservative to penetrate the coral tissues and then stored at -20°C until processing.

### Coral Genetic Identification

Of the 23 samples collected for microbiology, sufficient biomass remained in 19 samples for genetic analysis of the octocoral host (**Table 1**). Octocorals were identified to genus initially using a key based on morphological characters (Bayer, 1981) and subsequently using a genetic barcode commonly applied to the Octocorallia: the mitochondrial mismatch repair gene homolog, *mtMutS* in combination with *cox1* (McFadden et al., 2011). Total genomic DNA was extracted from octocoral tissue (whole polyps and adjacent coenenchyme) using a CTAB buffer and a single chloroform-only extraction method (Berntson et al., 2001; France, 2007), and quantified using the NanoDrop Lite Spectrophotometer (Thermo Scientific; USA).

PCR amplifications were conducted using GoTaq polymerase (Promega) and included 5X GoTaq^®^ Green buffer, 2 mM MgCl_2_, 0.4 μM dNTPs mix, 0.24 μM of each primer, 5 μg of bovine serum albumin (BSA, Sigma) and 1 U of GoTaq^®^ polymerase, with the following cycle profiles: for *mtMutS* [amplicon 825 base pair (bp) length] 95°C for 2 min, 35 cycles of 95°C for 20 s, 50°C for 30 s, 72°C for 50 s and a final step of 72°C for 5 min; for *cox1* (amplicon 990 bp length) 94°C for 3 min, 30 cycles of 94°C for 20 s, 46°C for 30 s, 72°C for 60 s and a final extension step of 72°C for 2 min.

All PCR amplified products were sent to Beckman Coulter Genomics (Beckman Coulter, Inc., Sykesville, MD, USA) for Sanger sequencing. Sequence traces were edited at the University of Louisiana, Lafayette using Sequencher version 4.6 (Gene Codes Corporation; USA) and then each sequence was queried against the Genbank nucleotide database using the BLASTn algorithm (NCBI, Altschul et al., 1990), and compared to unpublished data of colleagues working on similar taxa. Sequences were aligned to those from other octocoral taxa being worked on in the France lab or acquired from GenBank using Clustal W (Thompson et al., 1994) in the program BioEdit (Hall, 1998), and indels checked against the translated amino acid sequences to maintain reading frame. Aligned sequence datasets were then analyzed using the maximum likelihood method in RaxML 7.2.8 through the CIPRES web-portal (Stamatakis et al., 2008).

### Nucleic Acid Extraction

Two polyps (~50 mg) were removed from each coral sample using flame-sterilized forceps and dissecting shears. DNA was extracted using the MOBIO PowerPlant DNA Isolation Kit (MO BIO Laboratories; Carlsbad, CA, USA). Per Sunagawa et al. (2010), modifications to this protocol included the addition of lysozyme and extended incubation periods at room temperature 24 and 65°C. Samples were then homogenized using 400 mg each of sterile 0.1 and 0.5 mm zirconia/silica beads (BioSpec Products; Bartlesville, OK, USA) in a Mini-BeadBeater-1 (Biospec Products; Sunagawa et al., 2010). The bacterial and universal primers 63F (5′CAGGCCTAACACATGCAAGTC3′; IDT; Iowa City, IA, USA; Marchesi et al., 1998) and 1542R (5′AAGGAGGTGATCCAGCCGCA3′; IDT; Pantos et al., 2003) were used to screen the samples to confirm amplification of the target 16S bacterial rRNA genes, rather than the possible amplification of coral 18S ribosomal rRNA genes by polymerase chain reaction (Galkiewicz and Kellogg, 2008). DNA concentrations from the extraction were quantified for each sample using a Quant-iT^TM^ PicoGreen dsDNA Assay Kit (Invitrogen: Eugene, OR, USA) as outlined in the manufacturer’s protocol and sent for sequencing.

### 16S rRNA Gene Pyrosequencing

Unamplified DNA extracted from the samples was sequenced by 454 pyrosequencing (Selah Genomics; Greenville, SC, USA) using GS FLX Titanium chemistry and V4–V5 targeting primers following Roche 454’s standard protocol for amplicons (Claesson et al., 2010): forward primer (5′ AYTGGGYDTAAAGNG; IDT) and reverse primer (5′ CGTATCGCCTCCCTCGCGCCATCAG; IDT). Sequence data from all samples were deposited in the NCBI Sequence Read Archive (SRA) under Bioproject number PRJNA296835.

### Bioinformatics and Statistical Analysis

Analysis of the sequence data was conducted using the bioinformatics packages QIIME 1.5.0 on the Data Intensive Academic Grid (DIAG), a National Science Foundation funded MRI-R2 project #DBI-0959894, and QIIME 1.9.1 on the Amazon Elastic Compute Cloud (Amazon “EC2”; Caporaso et al., 2010). Our bioinformatics workflow and all resulting processed files are available online as a USGS data release (Kellogg and Lawler, 2015).

A total of 1,308,658 raw reads were generated from the 23 individual coral samples. Quality checks were performed using the split_libraries.py with the following parameters: sequence length (minimum sequence length of 200 bp and a maximum length of 700 bp), a minimum average quality score of 25, a maximum of one primer mismatch, and a maximum of a six homopolymer run (Kunin et al., 2010). SFF files were split into individual sample libraries based on the designated 10 bp identification barcode assigned to each sample during library preparation. The 889,914 sequences that passed the quality checks were then denoised using denoiser_preprocess.py, denoiser.py, and inflate_denoiser_results.py. This process was employed to reduce the number of erroneous operational taxonomic units (OTUs) and increase the accuracy of the sequence processing (Quince et al., 2011). Samples containing fewer than 10,000 sequences were removed prior to OTU selection to maximize the sequence data available. Furthermore, corals with no confirmed genetic identification were also removed at this stage, leaving a final total of 16 samples (**Table 1**, highlighted). Moving forward, OTUs were selected using an open-reference method (pick_open_reference_otus.py), with a 97% similarity threshold (Rideout et al., 2014). This method clustered sequences from each sample against the Greengenes reference database release 13_8 (DeSantis et al., 2006). Sequences that were not matched during the reference comparison were reevaluated using the *de novo* reference method. Sequences were then aligned using usearch (Edgar, 2010), which included the removal of chimeras. Representative OTU sequences (defined as one representative from each OTU) were selected, assigned a taxonomic classification (uclust; Edgar, 2010), and used to create a phylogenetic tree (Price et al., 2010). Sequences were then filtered to remove absolute singletons (defined as an OTU present only once in the analysis). Sequences classified as chloroplasts and mitochondria were removed from the OTU table as were any sequences classified as Eukarya or Archaea. Samples were then rarified to the number of sequences present in the smallest sample (10,333) before further diversity analysis was completed. Analysis of the core diversity associated with the coral species was completed using compute_core_diversity.py.

Alpha and beta diversity calculations as well as relative abundance summaries were conducted using alpha_diversity.py, beta_diversity.py, and summarize_taxa_through_plots.py. Alpha diversity metrics included Chao index (Chao, 1984), Shannon diversity index (Shannon, 1948), and Simpson diversity index (Simpson, 1949) (**Table 2**). These indices were employed to assess the richness and evenness of the associated microbiota within each individual sample. To assess beta diversity (similarities or differences across samples), three matrices were used based on phylogenetic and taxonomic relationships between sequences. Weighted and unweighted unit fraction (UniFrac; Lozupone and Knight, 2005) measurements were recorded to evaluate the importance of the presence/absence of specific taxa within the samples (unweighted Unifrac) compared to the abundance of these taxa (weighted Unifrac; Fukuyama et al., 2012). Bray–Curtis was also assessed to evaluate differences between each sample based on the number of sequences per OTUs. To visualize beta diversity, principal coordinate analysis (PCoA) plots were prepared in R-Studio (R Development Core Team, 2014) using the previously described metrics. In addition, pairwise analysis of similarities (ANOSIM) was performed to further examine the statistical variation between sample groups (e.g., environmental parameters, location, or species diversity; Chapman and Underwood, 1999). A similarity percentage (SIMPER) was also used to determine the key contributing families responsible for the observed patterns. This statistical analysis was completed using PRIMER-E Ltd (Clarke and Warwick, 2001) and R-Studio. Figures for this study were produced in R-Studio (R Development Core Team, 2014) using the vegan (Oksanen et al., 2015) and gplots packages (Warnes et al., 2015).

**Table 2:** Alpha diversity analysis of coral samples.

Corals	Canyons	Sample ID	No. Reads*	Operational taxonomic units (OTUs)	ACE richness	Chao1 richness	Shannon index	Simpson index	Simpson evenness
*Anthothela grandiflora*	Baltimore	NF.13Q6	29,474	43	69.70	62.13	1.53	0.52	0.048
*Anthothela grandiflora*	Baltimore	NF.13Q7	26,064	49	77.63	119.00	2.16	0.72	0.074
*Anthothela grandiflora*	Baltimore	NF.15Q6	19,005	40	82.93	97.75	1.59	0.61	0.064
*Anthothela grandiflora*	Baltimore	NF.15Q7	24,553	45	67.94	69.00	1.88	0.65	0.064
*Anthothela grandiflora*	Baltimore	NF.16Q7	25,060	42	81.75	105.33	1.61	0.54	0.052
*Anthothela grandiflora*	Baltimore	NF.17Q6	26,819	49	111.32	109.00	1.29	0.41	0.034
*Anthothela grandiflora*	Baltimore	NF.17Q7	10,333*	56	101.88	83.27	2.48	0.77	0.079
*Anthothela grandiflora*	Baltimore	NF.18Q7	11,969	95	210.69	209.833	2.51	0.76	0.044
*Anthothela grandiflora*	Baltimore	RB.686Q4	24,843	30	54.59	56.00	1.60	0.63	0.089
*Anthothela grandiflora*	Norfolk	RB.687Q5	14,269	66	135.65	109.50	2.52	0.76	0.062
*Anthothela grandiflora*	Norfolk	RB.688Q1	14,289	80	127.49	113.21	1.61	0.43	0.022
*Anthothela grandiflora*	Norfolk	RB.688Q5	298,193	77	115.09	110.00	1.90	0.55	0.029
*Anthothela* sp.	Baltimore	NF.18Q6	13,039	49	80.138	87.00	2.68	0.81	0.105
*Anthothela* sp.	Norfolk	RB.686Q5	32,479	67	96.61	101.50	2.38	0.74	0.067
*Anthothela* sp.	Norfolk	RB.688Q4	254,947	215	569.08	446.92	3.09	0.83	0.028
*Alcyonium grandiflorum*	Norfolk	RB.688Q3	13,216	423	454.32	457.44	5.54	0.87	0.018

*All samples were rarified to 10,333 sequences before diversity indices were calculated.

## Results

The octocoral hosts were initially identified as *A. grandiflora* based on morphology (to genus) and distribution (to species). Comparisons to the GenBank nucleotide database and unpublished data suggested these represented three different species: *A. grandiflora* (the most commonly collected Anthothelidae in both canyons and 12 of the 23 samples analyzed in this study), *Anthothela* sp. (*n* = 5), and “*Alcyonium” grandiflorum* (the genus name is shown here in quotes to denote an ongoing systematic revision which will see *Alcyonium grandiflorum* transferred to a new genus in the family Anthothelidae; pers. comm., K. Moore, Commonwealth Science and Industrial Research Organisation, Australia; *n* = 2; **Table 1**). Mitochondrial DNA sequences revealed three haplotypes using both *mtMutS* (GenBank accession numbers: *A. grandiflora*, KU712085; *Anthothela* sp. KU712082; *Al. grandiflorum*, KU712083 and KU712084) and *cox1* (GenBank accession numbers: *A. grandiflora*, KU712079; *Anthothela* sp. KU712080; *Al. grandiflorum*, KU712081). The *Al. grandiflorum* sequences are highly divergent from both *Anthothela* species (from *A. grandiflora* uncorrected *p* distance = 5% at *mtMutS* and *p* = 2% at *cox1*), but *Anthothela* sp. shows low sequence divergence from *A. grandiflora* (*p* distance < 0.2% at *mtMutS* and at *cox1*). Such a low level of interspecific divergence within a genus is not unusual for mitochondrial genes of octocorals (Pante and France, 2010; McFadden et al., 2011; Pante et al., 2015). Four of the 23 samples were not analyzed for coral genetics and were removed from our analysis (NF.01Q7, NF.02Q7, NF.16Q6, and RB.687Q4) although their bacterial sequence data are included in the SRA data file for completeness. In addition, samples with fewer than 10,000 sequence reads (NF.02Q7, RB.686Q2, RB.687Q3, and RB.688Q2) were removed before primary analysis to increase rarefaction depth. As such, 16 samples were analyzed further, nine from Baltimore Canyon (one *Anthothela* sp. and eight *A. grandiflora* samples) and seven from Norfolk Canyon (one *Al. grandiflorum*, two as *Anthothela sp*., and four *A. grandiflora*; **Table 1**, **Figure 1**).

### Alpha and Beta Measurements among Bacterial Diversity

**FIGURE 2:**
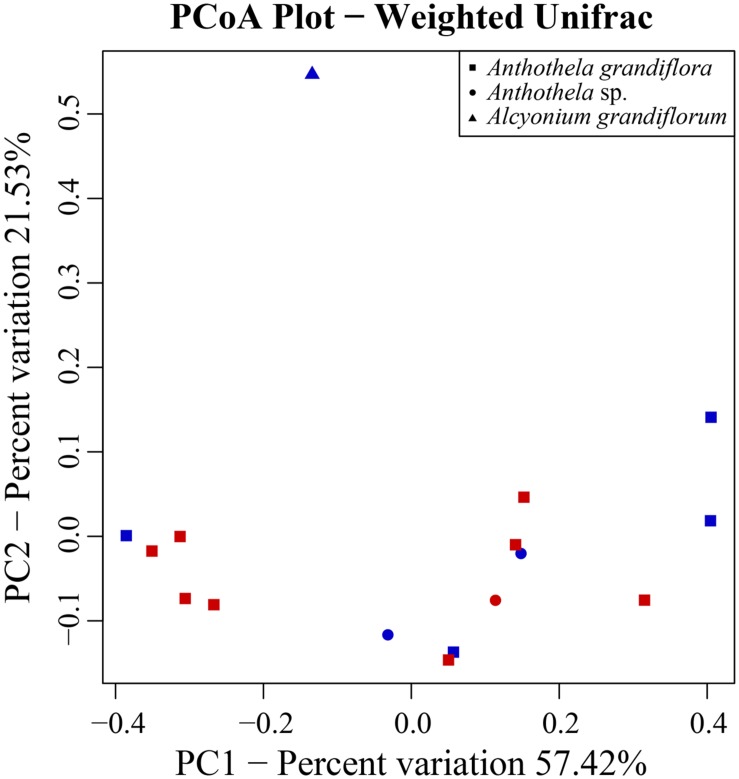
**Principal coordinate analysis (PCoA) plot of weighted Unifrac distance.** Principal coordinates analysis was used to plot the beta diversity of bacterial communities using the weighted Unifrac Matrix. Red symbols indicate samples collected from Baltimore Canyon, while blue symbols indicate samples collected from Norfolk Canyon. Symbol shapes defined in the legend distinguish the samples based on host genetic identification (e.g., *A. grandiflora*, *Anthothela* sp., and *Al. grandiflorum*).

Phylogenetic relationships samples (beta diversity) were compared using three primary diversity matrices (weighted UniFrac, unweighted UniFrac, and Bray–Curtis) and visualized using principal coordinates analysis (PCoA). Diversity of samples was first evaluated based on coral host. Here, samples within the genus *Anthothela* (*A. grandiflora* and *Anthothel*a sp.) clustered separately from *Al. grandiflorum*, accounting for ~57% of the statistical variation (**Figure 2**). Due to the small sample size (*n* = 1), *Al. grandiflorum* could not be included in the analysis of similarities (ANOSIM) to assess the correlation between bacterial diversity and coral species. Samples associated with *Anthothela* sp. and *A. grandiflora* were compared, revealing no significant difference between the two species (ANOSIM: *R* = 0.03, *p* = 0.27). Sample site was also assessed, indicating no significant correlation between coral-associated bacterial diversity and the canyon of origin (ANOSIM: *R* = -0.02, *p* = 0.45). The influence of depth of sample sites, water temperature, and salinity were also examined by PCoA, but were not correlated with bacterial diversity (results not shown). Similarity percentage analysis (SIMPER) was then used to examine representative bacterial taxa (family level) responsible for the differentiation between *Anthothela* samples and *Al. grandiflorum*. Overall, *Anthothela* bacterial communities had an average similarity of 63%. Considerable dissimilarity (~72%) was observed between *Al. grandiflorum* and *Anthothela* microbiomes. Contributing families included Shewanellaceae (~19%) and Pirellulaceae (~11%) which were only present in *Al. grandiflorum*. Unclassified Oceanospirillales (16%), unclassified Spirochaetales (12%), Spirochaetaceae (10%), and Colwelliaceae (10%) also defined the differences seen between *Anthothela* samples and *Al. grandiflorum*.

To measure the bacterial diversity present within each individual sample, a series of alpha diversity indices were used (**Table 2**). Shannon (Shannon, 1948), Simpson (Simpson, 1949), and Chao 1 (Chao, 1984) diversity indices account for evenness (defined as the abundance of species present) and richness (defined as the number of species or OTUs) as well as the total number of species observed. These measurements (Shannon = 5.54, Simpson = 0.87, and Chao 1 = 457.44) revealed greater species richness and evenness in the *Al. grandiflorum* sample compared to the rest of the *Anthothela* samples (**Table 2**). In general, Shannon measurements were fairly consistent across *Anthothela* samples (average Shannon = 2.27) with increased diversity in two samples (RB.688Q4 = 2.68 and NF.18Q6 = 3.09). Similar trends were seen in Chao 1 and Simpson measurements. To visualize the diversity driving these patterns, bacterial communities were characterized at the phylum, order, and family levels for each coral host.

### Bacterial Community Composition Associated with *Al. grandiflorum*

**FIGURE 3:**
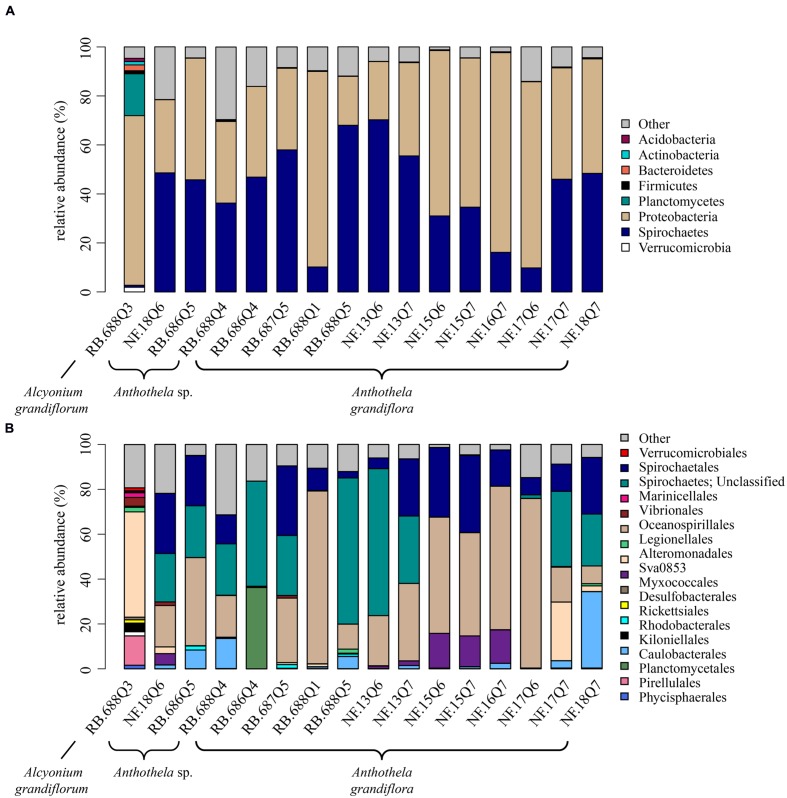
**Relative abundance of bacterial taxa in coral samples. (A)** Phyla present at ≥1% relative abundance in at least one sample. All remaining taxa are summarized under “Other.” **(B)** Orders present at ≥1% relative abundance in at least one sample. All remaining taxa are summarized under “Other.” Samples collected from Baltimore Canyon begin with the letters “NF” and those from Norfolk Canyon with “RB.”

Proteobacteria dominated the *Al. grandiflorum* sample (RB.688Q3), accounting for ~69% of the relative abundance with Planctomycetes representing the second most abundant at ~17% (**Figure 3A**). Additional minor contributors to the bacterial diversity included Bacteroidetes (~2%), Acidobacteria (~1%), Actinobacteria (~1%), Firmicutes (~1%), and Verrucomicrobia (~1%). Phyla representative of less than 1% of the relative abundance of the sample were labeled as “Other” (representing ~5% of the sample’s relative abundance); some of these included Chlamydiae, Deferribacteres, Lentisphaerae, and Nitrospira.

The bulk of the bacterial diversity distinguishable at the order level was found within *Al. grandiflorum* (**Figure 3B**). Alteromonadales dominated bacterial diversity at 47% relative abundance, followed by Pirellulales, accounting for 13% of the relative abundances. Additional contributing bacterial populations included α-proteobacteria (order Rhodobacterales), δ-proteobacteria (orders Desulfobacterales and Myxococcales), and γ-proteobacteria (orders Legionellales and Vibrionales). Rhodobacterales and Vibrionales accounted for approximately 4% of the relative abundances with Desulfobacterales, Myxococcales, Marinicellales, Phycisphaerales, and Planctomycetales observed in the *Al. grandiflorum* sample, but at smaller relative abundance (~2%). Three of these five proteobacterial orders were only present in *Al. grandiflorum*: Rhodobacterales, Desulfobacterales, and Myxococcales.

Within the order Alteromonadales, Shewanellaceae (~35%), and Colwelliaceae (~11%) were both found at high relative abundance. Additionally, Pirellulaceae, under the order Pirellulales, contributed ~13% of the relative abundance of this sample. Other contributing families present in less than 5% of the relative abundance of *Al. grandiflorum* included: Verrucomicrobiaceae, Spirochaetaceae, Planctomycetaceae, Pseudoaltermonadaceae, Marinicellaceae, and Rhodobacteraceae. The bacterial genus *Shewanella* accounted for ~35% relative abundance. Approximately 2% of the identifiable genera in *Al. grandiflorum* were named; these included *Phaeobacter* (family Rhodobacteraceae), *Planctomyces* (family Planctomycetaceae), and *Pseudoalteromonas* (family Pseudoalteromonadaceae). With the exception of *Pseudoalteromonas*, each was exclusively identified in *Al. grandiflorum*.

### Bacterial Community Composition of *Anthothela*

In total, 15 coral samples were classified under the genus *Anthothela*, consisting of both *A. grandiflora* (*n* = 12) and an unknown *Anthothela* species (*n* = 3). At the phylum level, roughly half of the 15 samples were dominated by Proteobacteria (~48% average relative abundance). The second most dominant bacterial group, Spirochaetes (**Figure 3A**), accounted for ~42% average relative abundance of the *Anthothela* samples (~43% in *Anthothela* sp. and ~41% in *A. grandiflora* samples). Unlike the *Al. grandiflorum* sample, Proteobacteria and Spirochaetes were the only bacterial groups discernible at the phylum level. Phyla representative of less than 1% of the relative abundance in at least one sample were labeled as “Other” (~10% of the total relative abundance). These included Chloroflexi, Lentisphaerae, and Nitrospira.

At the order level, all bacteria groups were classifiable with the exception of one, unclassified Spirochaetes (**Figure 3B**). Bacterial diversity associated with *Anthothela* genus samples (*A. grandiflora* and *Anthothela* sp.) varied slightly with key communities including: Oceanospirillales (34%), unclassified Spirochaetes (24%), and Spirochaetales (17%). Other orders were observed at higher abundance in several of the *Anthothela* samples: Alteromonadales accounted for 26% of *A. grandiflora* sample NF.17Q7; Deltaproteobacteria Sva0853 were present in 6 of the 15 samples, ranging from 1 to 16% relative abundance. Kiloniellales were visibly represented in 10 samples, ranging from 1 to 34% relative abundance. Caulobacterales, with ~36% relative abundance, were present in sample RB.686Q4. Bacterial groups observed in one or two of the *Anthothela* genus samples at lower relative abundance included: Rickettsiales present at ~2% in three Norfolk Canyon samples (RB.686Q5, RB.687Q5, and RB.688Q5); Legionellales accounting for ~2% of samples RB.688Q5 and NF.18Q7; and Vibrionales present at ~2% of samples RB.687Q5 and NF.18Q6. Three of the bacterial groups present in *Anthothela* samples were not observed in *Al. grandiflorum*: Caulobacterales, Kiloniellales, and Rickettsiales.

Families and identifiable genera (present at greater than 1% relative abundance in at least one sample) were also assessed for *Anthothela* samples. *Spirochaeta* (order Spirochaetales, family Spirochaetaceae) were the most abundant bacteria (3–34% relative abundance) observed in 13 of the 15 samples. The family identified as Endozoicomonaceae accounted for ~2% of two samples RB.687Q5 and RB.688Q1. Because we could not find this family defined in any taxonomic literature, sequences identified as Endozoicomonaceae were run through RDP Classifier (Wang et al., 2007) for further assessment. In Classifier, Endozoicomonaceae sequences were categorized as the family Hahellaceae. Lastly, *Moritella* sequences (order Alteromonadales, family Moritellaceae) were observed dominating *A. grandiflora* sample NF.17Q7, accounting for ~26% relative abundance.

### Core Microbiome

**FIGURE 4:**
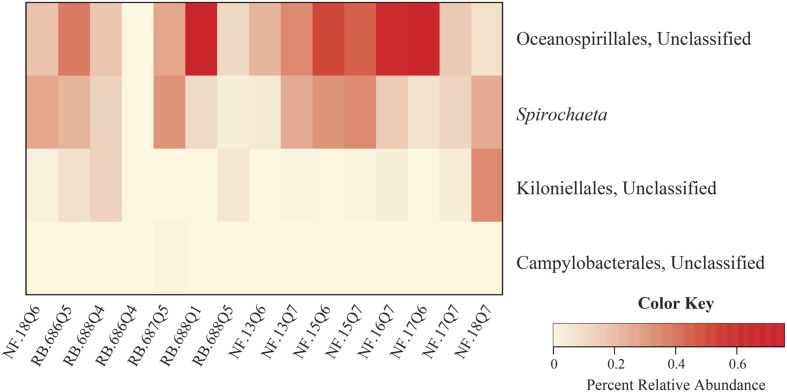
**Core microbiome of *Anthothela* samples.** A heatmap was created to visualize the core community of the 15 samples within the *Anthothela* genus. Bacterial taxa represented in this figure were unique to the *Anthothela* spp. core microbiome present in 90% of the samples.

To evaluate the potential conserved-core diversity, samples were first assessed at the level of family Anthothelidae, i.e., across all 16 samples. One identifiable genus, *Spirochaeta*, was observed in every sample. Next, we evaluated the core diversity at the *Anthothela* genus level (*n* = 15). Assessment of *Anthothela* samples revealed no additional unique taxa, with *Spirochaeta* still the only shared taxon. From here, conserved bacteria were assessed at 90% sample coverage revealing four conserved communities: unclassified orders (Oceanospirillales, Kiloniellales, and Campylobacterales) and genus *Spirochaeta* (**Figure 4**). Individual species were examined at 100% sample coverage: *A. grandiflora* (*n* = 12) and *Anthothela* sp. (*n* = 3). *A. grandiflora* samples only shared the genus *Spirochaeta*, while *Anthothela* sp. included the genera *Propionibacterium*, *Pseudoalteromonas* as well as unclassified bacteria within Spirochaetes, Kiloniellales, Campylobacterales, Oceanospirillales, and Brachyspiraceae.

## Discussion

Relatively little is known about cold-water coral microbiomes in comparison to those of tropical corals. Prior to this study no microbial assessment had been completed on cold-water Anthothelidae corals. Because some tropical coral species have shown correlation between their bacterial composition and environmental parameters (e.g., geographic location, depth, ambient water-temperature, and surrounding organisms; Littman et al., 2009; Barott et al., 2011), similar relationships were anticipated within the host–microbe interactions of the cold-water corals collected during this study. However, bacterial composition of samples was not found to be significantly different based on canyon of origin (**Figure 2**). Beta diversity matrices indicated relationships between the taxa present and their abundance drove the diversity. Alpha diversity measurements also indicated higher bacterial diversity in the *Al. grandiflorum* sample (**Table 2**). This as well as the ANOSIM results further supported the PCoA distribution showing the clustering of all *Anthothela* samples separate from *Al. grandiflorum* (RB.688Q3).

### Proteobacteria

In most of the marine environment, Proteobacteria dominate the bacterial diversity (Amaral-Zettler et al., 2010). This is true for many tropical coral species (Frias-Lopez et al., 2002; Bourne and Munn, 2005; Thurber et al., 2009) as well as cold-water scleractinians and octocorals (Penn et al., 2006; Yakimov et al., 2006; Neulinger et al., 2008; Hansson et al., 2009; Galkiewicz et al., 2011; Van Bleijswijk et al., 2015). In this study a similar pattern was observed. Proteobacteria, primarily the orders Oceanospirillales, Kiloniellales, and Alteromonadales accounted for the majority of the diversity in samples. Other minor contributing Proteobacteria included Rhodobacterales, Desulfobacterales, Myxococcales, Legionellales, and Vibrionales.

#### Proteobacteria in *Al. grandiflorum*

Alteromonadales (specifically families Shewanellaceae and Colwelliaceae) were observed as the dominant order within *Al. grandiflorum*. Genera classified under these families are ubiquitous throughout marine environments, described in tropical (Bourne and Munn, 2005; Ritchie, 2006; Thompson et al., 2006; Shnit-Orland and Kushmaro, 2009; Shnit-Orland et al., 2010), temperate (La Rivière et al., 2015), and cold-water coral species (Kellogg, 2008; Kellogg et al., 2009; Galkiewicz et al., 2011; Gray et al., 2011). Their presence in cold-water environments was expected as many members of both Shewanellaceae and Colwelliaceae are psychrophilic (organisms capable of growing in cold, extreme environments; Bowman, 2014; Satomi, 2014). Biochemical properties of Colwelliaceae members include energy production through the decomposition of organic matter and nitrate reduction (Bowman, 2014). Species classified under the genus *Shewanella* (family Shewanellaceae) are generally Gram-negative, facultative anaerobes, recognized for their roles in nitrate and iron reduction (Hau and Gralnick, 2007; Coursolle and Gralnick, 2012; Kim et al., 2012; Satomi, 2014). Prevalent throughout the marine environment, members of this family have been isolated from numerous deep-sea and cold-water environments including the Marianas Trench (Kato et al., 1998) and the Arctic Ocean (Kim et al., 2012). *Shewanella* isolated from the mucus of tropical coral genus *Favia* demonstrated both antibacterial properties and antibiotic resistance (Shnit-Orland and Kushmaro, 2009; Shnit-Orland et al., 2010). These beneficial or mutualistic relationships are thought to contribute to the overall health of the host organism, providing protection from pathogenic or opportunistic bacteria.

Members of the order Rhodobacterales, specifically the genus *Rhodobacter* are thought to be among the most abundant, diverse, and metabolically influential bacteria within the marine environment (accounting for ~25% of the total bacteria present in coastal and polar regions; Wagner-Dobler and Biebl, 2006). Rhodobacterales are found ubiquitously amongst coral reef systems, typically observed in surface waters, reef invertebrates, and their associated biofilms (Galkiewicz and Kellogg, 2008; Sunagawa et al., 2009; Sharp et al., 2012; Roder et al., 2014; La Rivière et al., 2015). Functionally these bacteria are diverse, capable of contributing to the reduction of trace metals and the production of antibiotic compounds (Brinkhoff et al., 2004). Other notable members also contribute to the global carbon and sulfur cycles through the oxidization of carbon monoxide and production of dimethylsulfide (Wagner-Dobler and Biebl, 2006). To date a few Rhodobacterales (particularly members of the family Rhodobacteraceae) have been identified in cold-water coral species: *Al. digitatum* (Alsmark et al., 2012) and *L. pertusa* (Neulinger et al., 2008; Kellogg et al., 2009).

Desulfobacterales (class α-proteobacteria) are documented sulfate-reducing bacteria, functionally described as hydrogenotrophs (capable of using H_2_ as an energy source within the metabolic pathway; Kuever et al., 2005; Kimes et al., 2010). These members appear to be common within deep-sea ecosystems, including microbial mats (Burow et al., 2014), sediments (Simister et al., 2015), seeps (Jaekel et al., 2013), and one unidentified cold-water coral (Simister et al., 2015). In contrast, Myxococcales (class α-proteobacteria) are commonly observed in terrestrial environments, with some present in marine ecosystems (though, notably less; Reichenbach and Dworkin, 1992). Several have been identified in coral species including *Eunicella cavolini* (Bayer et al., 2013a) and *Mussismilia braziliensis* (Garcia et al., 2013). Functionally these bacteria may contribute to the production of nutrients through sulfate reduction and the decomposition of organic matter (Reichenbach, 1999; Baker et al., 2015).

Legionellales and Vibrionales were each identified as a small percentage (~2%) of the bacterial community associated with *Al. grandiflorum*. Legionellales, described as facultative or obligate intracellular parasites are recognized for infecting invertebrate and vertebrate species (Garrity et al., 2005). While commonly associated with marine environments, members of the Legionellales have rarely been identified in corals (tropical or cold-water; Meron et al., 2012; Ransome et al., 2014). Unlike Legionellales, *Vibrio* spp. (order Vibrionales) are common associates of shallow-water corals (Kushmaro et al., 2001; Rohwer et al., 2001; Ben-Haim et al., 2003a; Bourne and Munn, 2005; Lampert et al., 2006; Ritchie, 2006; Rosenberg et al., 2007) and have also been found in association with the cold-water coral species *L. pertusa* (Neulinger et al., 2008; Galkiewicz et al., 2011), *E. verrucosa* (Hall-Spencer et al., 2007), *P. arborea*, *P. superba*, and *C. koolsae* (Gray et al., 2011). Although, these bacteria are primarily acknowledged for their roles as opportunistic or pathogenic bacteria associated with coral disease and bleaching events (Toren et al., 1998; Ben-Haim et al., 2003a,b; Hall-Spencer et al., 2007; Sussman et al., 2008; Sweet and Bythell, 2012), they are also recognized as common members of healthy coral microbiomes (Bourne and Munn, 2005; Raina et al., 2009; Ainsworth et al., 2015).

#### Proteobacteria in *Anthothela* Samples

Oceanospirillales was identified as one of the dominant bacterial groups in *Anthothela* samples, present in eight samples at over 20% relative abundance. Functional characteristics that have been associated with Oceanospirillales members include carbon fixation, sulfur oxidation, and biofilm production in the presence of trace metals, such as copper (Little et al., 1996; Swan et al., 2011; Speck and Donachie, 2012). These roles define both the acquisition of nutrients and potential attraction or inhibition of bacterial colonization within the host. In this study, Oceanospirillales were further classified to the family level, with identifiable groups consisting of unclassified Oceanospirillales and Endozoicomonaceae. At present time, it is unclear if Endozoicomonaceae is an accepted taxon, since those same sequences were identified by RDP Classifier as Hahellaceae with 99% confidence. With that in mind, members of the family Hahellaceae (specifically genus *Endozoicomonas*) are widespread in the marine environment. Numerous studies have assessed *Endozoicomonas* in tropical and temperate corals (Sunagawa et al., 2009, 2010; Yang et al., 2010; Cardenas et al., 2012; Lee et al., 2012; Morrow et al., 2012; Apprill et al., 2013; Bayer et al., 2013a,b; Carlos et al., 2013; Correa et al., 2013; Jessen et al., 2013; La Rivière et al., 2013; Pike et al., 2013; Ransome et al., 2014; Roder et al., 2015), as well as sea slug (Kurahashi and Yokota, 2007) and sponge species (Nishijima et al., 2013; Rua et al., 2014). Because *Endozoicomonas* are both common and highly abundant in healthy tropical corals species, relationships between these bacteria and their hosts have been thoroughly examined. Functional characteristics include nitrate reduction, chemotactic activity, and production of antimicrobial compounds (Kurahashi and Yokota, 2007; Rua et al., 2014; Tout et al., 2015). While members of the family Hahellaceae are common in tropical and temperate environments, they do not appear to be common in deep-sea corals. Few studies have observed bacteria classified under Hahellaceae in deep (>100 m) cold-water corals (Hansson et al., 2009; Kellogg et al., 2009; Van Bleijswijk et al., 2015). The symbiotic relationships between Hahellaceae and zooxanthellae are thought to be one of the driving influences of their abundance and presence in tropical corals (Pantos et al., 2015). The lack of algal symbionts in deep-sea corals may be why there are few Hahellaceae bacteria, if present at all, in these holobionts. In this study, members of the Hahellaceae (originally classified as Endozoicomonaceae) represent a small minority of the Oceanospirillales present (~2% in *A. grandiflora* samples RB.687Q5 and RB.688Q1).

Similar to Oceanospirillales, members of the order Kiloniellales were present in multiple *Anthothela* samples at relatively high abundance. Kiloniellales bacteria have been observed in several tropical coral species (Sharp et al., 2012; Soffer et al., 2015) as well as mussels (Cleary et al., 2015), sponges (Cleary et al., 2013), and algae (Wiese et al., 2009). Soffer et al. (2015), identified members of the order Kiloniellales at higher abundances in healthy coral *Orbicella annularis* than in diseased colonies, suggesting association in a beneficial capacity. Functionally, these chemoheterotrophic bacteria have been found to utilize nitrates within the metabolic process through denitrification (Wiese et al., 2009; Imhoff and Wiese, 2014).

Several contributing Proteobacteria were identified in individual *Anthothela* samples including Alteromondales, Caulobacterales, and Rickettsiales. Genus *Moritella* (order Alteromondales, family Moritellaceae) was present in *A. grandiflora* sample NF.17Q7 accounting for ~26% relative abundance. This bacterial group is specific to marine environments and generally classified as halophilic facultative anaerobes (Stanley et al., 2005; Urakawa, 2014). *Moritella* isolates have been collected from a wide variety of environments ranging from deep-sea sediments (Kato et al., 1998; Nogi et al., 1998; Xu, 2003) to tropical corals (Rohwer et al., 2001; Bourne, 2005; Bourne and Munn, 2005). In cold-water coral species, *Moritella* sequences have been described in the scleractinian *L. pertusa* collected from the Gulf of Mexico (Kellogg, 2008). In the individual *A. grandiflora* sample RB.686Q4, members of the Caulobacterales were found at relatively high abundance (~30%). Recognized for their unique morphology, members of this order contain a stalk-like flagellum utilized for adhesion to adjacent surfaces, including but not limited to host organisms (Starr and Skerman, 1965). While these bacteria tend to exhibit parasitic tendencies, they have been described as facultative commensals, potentially contributing to the acquisition of nutrients through their roles in carbon cycling (Abraham et al., 1999). These free-living bacterial communities are often found throughout the water column and have been observed in several tropical corals including the gorgonian *Pseudopterogorgia elisabethae* (Correa et al., 2013) and acroporid species (*A. granulosa*, *A. valida*, and *A. millepora*; Littman et al., 2009; Ainsworth et al., 2015). While members of the group Caulobacterales have been identified in deep ocean waters (Eloe et al., 2011), no prior studies have observed Caulobacterales associated with cold-water corals.

Members of the order Rickettsiales have previously been described as opportunistic, pathogenic, and/or associated with diseased tropical corals (Peters et al., 1983; Casas et al., 2004; Garcia et al., 2013; Miller et al., 2014; Peters, 2014). While they are common in tropical corals, Rickettsiales have currently been identified in one cold-water octocoral species *C. koolsae* (Gray et al., 2011). In the present study, Rickettsiales were observed at low relative abundance (~2%) in only three Norfolk Canyon samples (RB.686Q5, RB.687Q5, and RB.688Q5). Because their functional characteristics are generally driven by ambient environmental fluctuations (e.g., increases in temperature and light intensity; Peters, 2014; Gignoux-Wolfsohn and Vollmer, 2015), we speculate that the opportunistic tendencies exhibited in tropical environments differ from those in cold-water environments. In this case, additional research is necessary to definitively assess the potential pathogenicity and overall functionality of these bacteria within the cold-water ecosystem.

### Spirochaetes

Spirochaetes were observed as one of the primary bacteria, dominating over half of the *Anthothela* samples. Spirochaetes are recognized as motile free-living, facultative/obligate anaerobes (Leschine et al., 2006). Functional characteristics displayed by members of this phylum include nitrogen and carbon fixation, as well as chemotactic responses to chemical stimulants (Greenberg and Canale-Parol, 1977; Kimes et al., 2010; Baker et al., 2015; Lilburn et al., 2015). Spirochaetes are commonly found in association with invertebrates at high abundance, including species of termites (Breznak, 2002), oligochaetes (Blazejak et al., 2005), sponges (Taylor et al., 2005), and tropical corals (Casas et al., 2004; Kimes et al., 2010, 2013; Closek et al., 2014). Previous studies using clone libraries have observed Spirochaetes in association with some cold-water corals (Penn et al., 2006; Kellogg et al., 2009; Gray et al., 2011), however, they have never been identified as a dominant member of the associated bacterial community. In this study, Spirochaetes were recognized as the prevailing phyla in roughly half of the total *Anthothela* samples (*n* = 15).

In *Anthothela* samples (*A. grandiflora* and *Anthothela* sp.), genus *Spirochaeta* (phylum Spirochaetes) continued to dominate the bacterial groups. New representative *Spirochaeta* sequences from this study were compared to those of environmental and invertebrate studies where *Spirochaeta* sequences had been observed. Sequences associated with *Anthothela* samples were most closely related to sequences isolated from deep-sea water (Accession KF758585, *E*-value of 7e^-165^) and microbial mats (Accession DQ218325, *E*-value of 1e^-161^). While sequences from *Anthothela* corals were not closely related to those of other coral species, presence of this bacterium across all 16 samples suggests conservation at the family level. Additionally, presence of this bacterium at such a high abundance, as observed in *Anthothela* samples, suggests a unique microbe–host interaction specific to that coral genus.

### Core Microbiome

In this study we applied a stringent approach to examine “core” conserved communities, evaluating bacterial groups present at the family (Anthothelidae), *Anthothela* genus, and individual species (*A. grandiflora* and *Anthothela* sp.) levels. To begin, samples were evaluated at the family and genus levels at 100% sample coverage, revealing one core-conserved bacterium present across all samples, a member of the genus *Spirochaeta*. As previously described, members of the phylum Spirochaetes are common throughout coral species, both tropical and cold-water (Casas et al., 2004; Penn et al., 2006; Kellogg et al., 2009; Gray et al., 2011; Kimes et al., 2013; Closek et al., 2014). These free-living nonpathogenic anaerobes are known to be capable of carbon fixation and organic carbon degradation (Baker et al., 2015). In this study, *Spirochaeta* was found to be one of the dominant bacterial groups, accounting for roughly 16% of the total relative abundance, thus suggesting a potentially important role within the coral microbiome.

Samples were then assessed at 90% sample coverage, particularly looking for conserved bacteria specific to *Anthothela* samples (**Figure 4**). In addition to the genus *Spirochaeta*, OTUs classified under the phylum Proteobacteria (orders; Oceanospirillales, Kiloniellales, and Campylobacterales) were identified. Previous studies described members of the orders Oceanospirillales and Kiloniellales as beneficial bacteria, contributing to their host system through the production of biofilm and antibacterial properties, respectively (Little et al., 1996; Swan et al., 2011). Oceanospirillales members have also been recognized for their influence in nutrient dynamics within the coral holobiont through the formation of dissolved inorganic materials produced during carbon fixation and sulfur oxidation (Swan et al., 2011). In contrast to Oceanospirillales and Kiloniellales, members of the order Campylobacterales are most commonly known for their association with coral disease (Frias-Lopez et al., 2002; Sunagawa et al., 2009; Sweet and Bythell, 2012; Vezzulli et al., 2013; Gignoux-Wolfsohn and Vollmer, 2015), but are also present in healthy corals (Sharp et al., 2012). Functionally these bacteria are well-known for their metabolic influence in nitrogen cycling and communication through bacterial quorum sensing (Kern and Simon, 2009; Golz et al., 2012).

**FIGURE 5:**
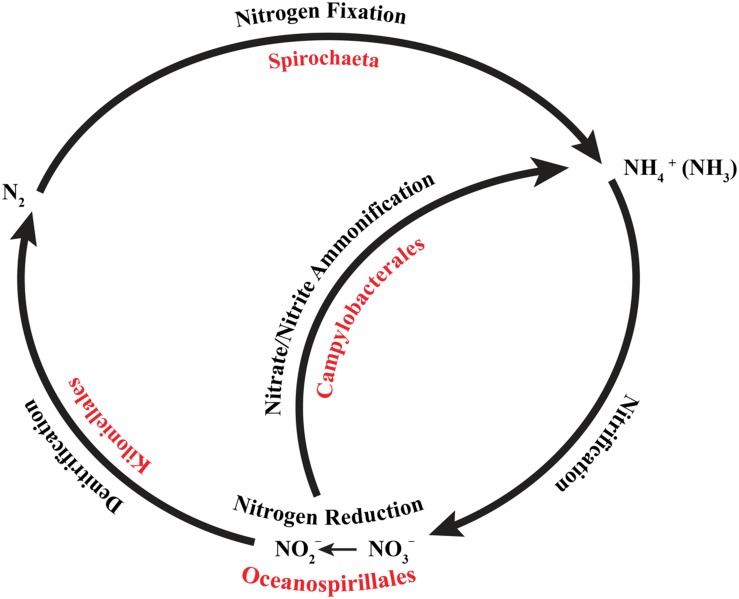
**Core bacterial groups potential roles within the nitrogen cycle.** Each of the bacterial groups present within the core microbiome of *Anthothela* samples was previously recognized for their roles within the nitrogen cycle. This diagram illustrates a simplified overview of the bacterial groups with their possible functions. This figure was adapted from one presented in Wegley et al. (2007).

Microbial functional genes capable of completing, or nearly completing, the nitrogen cycle have been identified in Pacific (Wegley et al., 2007) and Atlantic (Kimes et al., 2010) tropical corals. A complete nitrogen cycle has been shown via isotope incorporation in the cold-water coral *L. pertusa*, including ammonium production and assimilation, nitrification, denitrification, and nitrogen fixation (Middelburg et al., 2015), however, these roles have not been definitively associated to specific microbial symbionts of the coral yet. Based on previous descriptions of nitrogen-cycling abilities in the bacterial taxa that we observed in the conserved core of *Anthothela* spp., we have inferred a nearly complete nitrogen cycle (**Figure 5**). Members of the genus *Spirochaeta*, identified as the most conserved bacterium, were previously recognized for their roles in nitrogen fixation (Lilburn et al., 2015). During nitrogen fixation, nitrogen gas (N_2_) is converted to readily available organic compounds that may then be taken in (Zehr and Kudela, 2011). Nitrogen fixation contributes up to 1/3 of the nitrogen requirement in *L. pertusa* (Middelburg et al., 2015), supplementing organic nitrogen obtained by capture feeding (Duineveld et al., 2012; Mueller et al., 2014). Several members of the order Campylobacterales were documented for their contributions through nitrate ammonification (Tiedje, 1988). This is the process by which nitrate is converted to ammonium, thereby recycling nitrogen back into the system (Tiedje, 1988; Simon, 2002; Rädecker et al., 2015). In the order Oceanospirillales, members have been described for their contribution as nitrate reducers, reducing nitrate to nitrite (Zehr and Kudela, 2011). Lastly, members of the order Kiloniellales, classified as chemoheterotrophic aerobic bacteria, have shown potential in the processing of molecular nitrogen through denitrification (Imhoff and Wiese, 2014). In this process, nitrates are reduced back into N_2_ (dinitrogen) to be utilized by nitrogen-fixing bacteria. While likely important for the functioning of the *Anthothela* coral holobiont, the rate of nitrogen fixation observed in *L. pertusa* was small compared to the rates occurring in the open ocean, suggesting that cold-water corals do not significantly impact nitrogen cycling in the deep sea (Middelburg et al., 2015). This is in line with research that ascribed a similarly minor role in the nitrogen budget of coral reef ecosystems to tropical corals (Cardini et al., 2014).

Although the core microbiome of the *Al. grandiflorum* (*n* = 1) could not be evaluated, its bacterial community was assessed for possible functional characteristics associated with the biochemical processing of nitrogen. Similar to *Anthothela* samples, core members associated with *Al. grandiflorum* were found to belong to taxa previously documented for their metabolic abilities in nitrogen cycling. In addition to the potentially nitrogen-fixing *Spirochaeta* also seen in *Anthothela* spp., bacteria classified under families Shewanellaceae and Colwelliaceae (order Alteromonadales) have been acknowledged for their roles as nitrate reducers (Satomi, 2014; Lilburn et al., 2015). Classified as anaerobic ammonia-oxidizing bacteria, Pirellulales (phylum Planctomycetes) were thought to contribute through the removal of metabolic waste within the host microbiome (Mohamed et al., 2010). One of the end products of nitrate ammonification, ammonia, may be taken in by ammonia-oxidizing bacteria such as Pirellulales, resulting in the oxidization of ammonium and formation of nitrites (Zehr and Kudela, 2011). While members of the order Pirellulales and families Shewanellaceae and Colwelliaceae only show potential metabolic properties for a portion of the nitrogen cycle, other bacterial members may be present within *Al. grandiflorum* that complete the remaining functions. Further research using targeted functional genes (e.g., *nifH*, *amoA*), metagenomics, and transcriptomics are required to link these microbial roles definitively to specific bacterial community members.

## Conclusion

Our study provides insight into the previously uncharacterized microbiome of cold-water octocorals classified under the family Anthothelidae. The two *Anthothela* species (*A. grandiflora* and *Anthothela* sp.) shared similar bacterial communities, in contrast to *Al. grandiflorum* which had a highly diverse microbiome distinct from the rest. The bacterial communities become more different as the coral taxa are more distantly related. Additionally, the bacterial composition revealed no differentiation due to geographic location (canyon of origin). Evaluations of the core microbiome at 90% revealed a conserved bacterial community associated with the *Anthothela* genus. Many of the core bacteria share potential metabolic functions associated with nutrient provision and properties aiding in the protection of the coral host. Overall health and proliferation of cold-water corals are dependent on capture feeding and the presence of microorganisms (Duineveld et al., 2004; Roberts et al., 2006). Members of the core microbiome of *Anthothela* samples were recognized for their potential roles in the uptake and remineralization of organic and inorganic material. More specifically, many of the bacterial groups present were previously documented for their roles in nitrogen cycling, including nitrogen fixation (*Spirochaeta*), nitrate ammonification (Campylobacterales), nitrate reduction (Oceanospirillales), and denitrification (Kiloniellales). Additionally, *Al. grandiflorum* also contained a microbial community potentially capable of various functions specific to nitrogen cycling: nitrogen fixation, nitrate reduction, and ammonia oxidation. Unfortunately, with a sample size of *n* = 1, this evaluation is only a partial representation of the overall bacterial and functional diversity present within this species. Further research is necessary to investigate the microbial–host interactions, specifically the functionality of these bacterial associates and their role within the cold-water coral holobionts.

## Author Contributions

SL performed the DNA extractions, analysis of results, and writing of the manuscript. CK planned the experimental design and participated in the manuscript writing/editing and data analysis. SF and RC completed coral genetics and provided detailed methods and results for this analysis. SR and SB planned the research cruises, acquired samples and multibeam data, and edited the manuscript.

## Conflict of Interest Statement

The authors declare that the research was conducted in the absence of any commercial or financial relationships that could be construed as a potential conflict of interest.
